# Cell survival and DNA damage repair are promoted in the human blood thanatotranscriptome shortly after death

**DOI:** 10.1038/s41598-021-96095-z

**Published:** 2021-08-16

**Authors:** Laura G. Antiga, Lode Sibbens, Yasmina Abakkouy, Ronny Decorte, Wouter Van Den Bogaert, Wim Van de Voorde, Bram Bekaert

**Affiliations:** 1 Forensic Biomedical Sciences, Department of Imaging and Pathology, KU Leuven, Herestraat 49, Box 7003 71, 3000 Leuven, Belgium; 2 Department of Experimental and Health Sciences (CEXS), University Pompeu Fabra (UPF), Barcelona, Spain; 3 Laboratory of Forensic Genetics, UZ Leuven, 3000 Leuven, Belgium

**Keywords:** Genetics research, Gene expression, Gene regulation, Genomics

## Abstract

RNA analysis of post-mortem tissues, or thanatotranscriptomics, has become a topic of interest in forensic science due to the essential information it can provide in forensic investigations. Several studies have previously investigated the effect of death on gene transcription, but it has never been conducted with samples of the same individual. For the first time, a longitudinal mRNA expression analysis study was performed with post-mortem human blood samples from individuals with a known time of death. The results reveal that, after death, two clearly differentiated groups of up- and down-regulated genes can be detected. Pathway analysis suggests active processes that promote cell survival and DNA damage repair, rather than passive degradation, are the source of early post-mortem changes of gene expression in blood. In addition, a generalized linear model with an elastic net restriction predicted post-mortem interval with a root mean square error of 4.75 h. In conclusion, we demonstrate that post-mortem gene expression data can be used as biomarkers to estimate the post-mortem interval though further validation using independent sample sets is required before use in forensic casework.

## Introduction

Research into forensic transcriptome profiling has seen an exponential growth over the past decade and studies have demonstrated an increasing number of applications (for a review see Salzmann et al.^1^). Body fluid tests based on RNA markers have been successfully implemented as routine testing methods in forensic laboratories and provide additional information in cases where the source of the cell could be crucial^2^ while applications such as the determination of the age of stains^3^, the age of the donor^4^ and wound healing^5^ are also of interest to the community. RNA analysis of postmortem (PM) tissues, also called thanatotranscriptomics, can be used for multiple forensic purposes such as organ identification^6–9^ and post-mortem interval (PMI) estimation. In forensic medicine, the PMI is the time elapsed from the Time of Death (ToD) of an individual until the discovery of the body. Time of death estimation from PMI is a daily task of forensic pathologists, most crucial when a possible murder victim is found. In those cases, there is a need to estimate ToD quickly and accurately, since it can determine the course and success of a criminal investigation^10,11^.

Studies using mouse, zebrafish and human tissue samples from Genotype-Tissue Expression (GTEx) database suggest that some biological processes (BP) are still active up to 48 h after the death of an individual^12–14^, either because of the activation of regulatory genes that allow the transcription of genes that were not active before^13^ or because of gene regulation through induced changes in chromatin structure^12^. Other studies involving human tissue samples have demonstrated a bi-modal wave in PM prostate tissues initiated by over-expressed anti-apoptotic genes followed by apoptosis inducing genes^15^ or the simultaneous up-regulation of pro-apoptotic and down-regulation of anti-apoptotic genes in PM liver tissues^16^. Such studies have provided insight into PM gene regulation and have demonstrated the potential of the analysis of PM gene expression in forensic cases.

Exploiting PM processes have been the basis for PMI predictions in forensic investigations. The most widespread techniques are based on *algor*, *rigor* and *livor mortis*^17,18^. As these methods are based on the physiological state of a body the accuracy is dependent on the degree of body decomposition. At the same time, the body decomposition rate is affected by many factors, such as environmental conditions, insects, body weight or clothing^19^. Furthermore, the longer the PMI, the higher the inaccuracy produced by any ToD prediction method (e.g.: using the body temperature of a corpse is only useful until it reaches the ambient temperature^20^). An alternative, molecular method has been on the wish list of forensic scientists for decades. In particular RNA degradation has been a topic of interest for predicting PMI using the expression patterns of single genes (for reviews see^1,21,22^). For example, RNA degradation as measured through the expression levels of fatty acid synthase-messenger RNA (*FASN*) in whole blood and brain samples showed a significant correlation with the storage interval of the blood samples and with PMI in autopsy cases^23^. In another study a PMI prediction model was developed based on $\beta$-actin gene expression in rat brain while taking temperature factors into account^24^. Partemi and colleagues, on the other hand, showed that transcript expression of *GUSB*, *COL1A1* and *COLIII* in the human heart was independent from PMI while *NOS3* gene expression was found to be down-regulated with longer PMI (> 24 h)^25^.

The current study is, to our knowledge, the first to perform a transcriptomic analysis on blood obtained from a longitudinal sampling procedure on deceased human individuals. The aims of this study were to (1) identify up or downward clusters or patterns in the human blood thanatotranscriptome; (2) perform gene ontology (GO) analysis on the identified gene clusters; and (3) to develop a PMI prediction model using generalized linear regression based on PM gene expression patterns.

**Figure 1:**
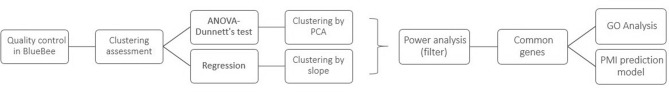
General pipeline of the study. After quality control and filtering of the initial transcripts, different methods were used to check data clustering. ANOVA-Dunnett’s tests and a regression analysis were performed in parallel to further analyze the 2 clusters of up- and down-regulated genes. Results were filtered with a power analysis and the common genes identified with both methods were then used as input for a GO enrichment analysis and to train a PMI prediction model.

**Figure 2:**
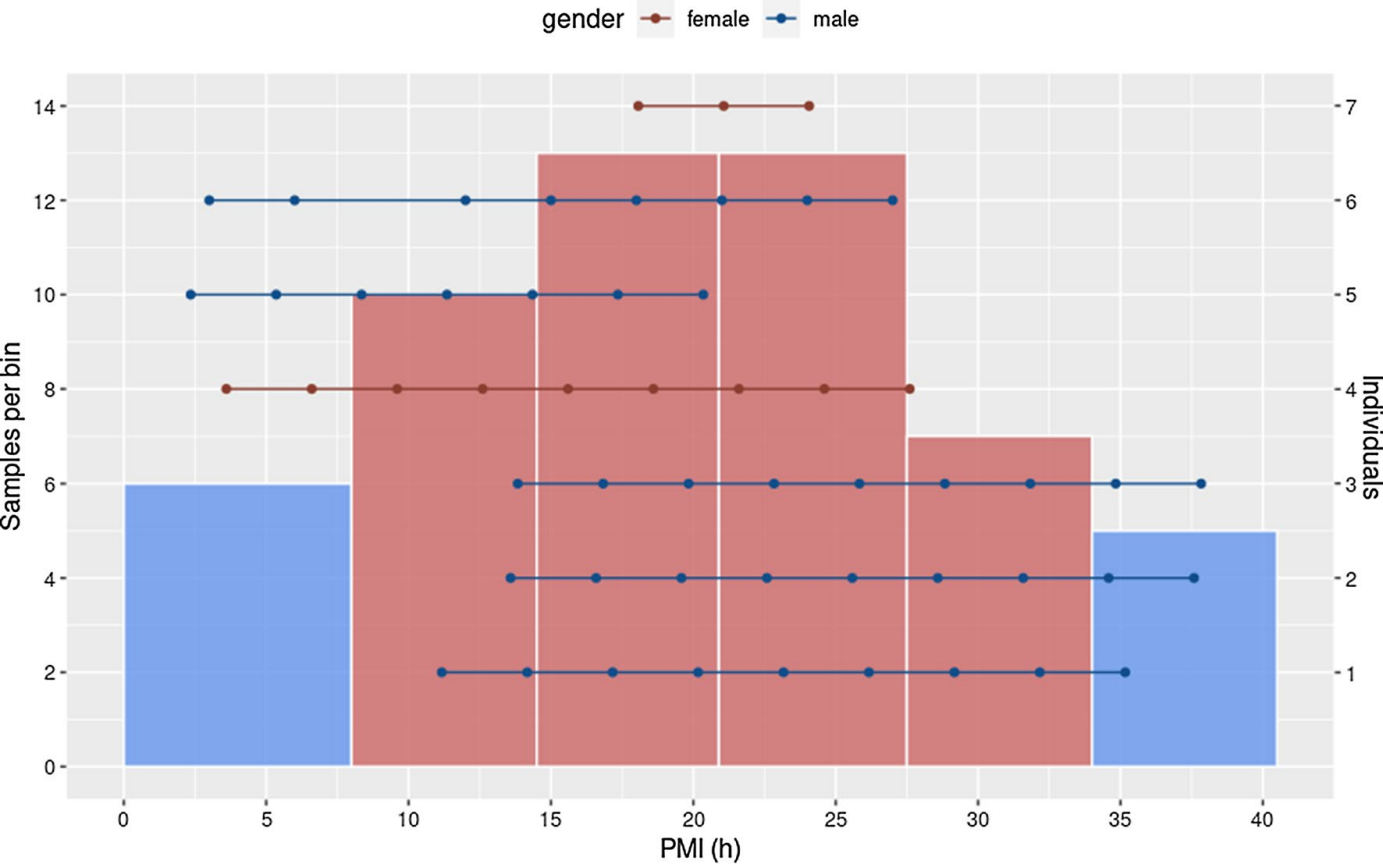
Dotplot of the sample distribution, per individual, over the time period covered. Each horizontal line represents a single individual (right y-axis), and each dot, a sample. The histogram represents the number of samples per bin in the ANOVA-Dunnett analysis. The early and late PMI bins are indicated in blue.

## Results

A summary of the analysis pipeline can be found in Fig. 1. A total of 54 samples were collected from 7 individuals, 5 men and 2 women with ages ranging from 56 to 89 years old and PMI ranging from 2 h 21 min to 37 h 50 min (Fig. 2). The samples were distributed into bins in order to compare the mean expression values of 10,635 RNA transcripts of each postmortem blood sample. Each bin spanned 6 h, except for the first bin, which covered an interval of 8 h. The number of samples in each bin, from lowest to highest PMI were 6, 10, 13, 13, 7 and 5 samples, respectively (Fig. 2).

**Figure 3:**
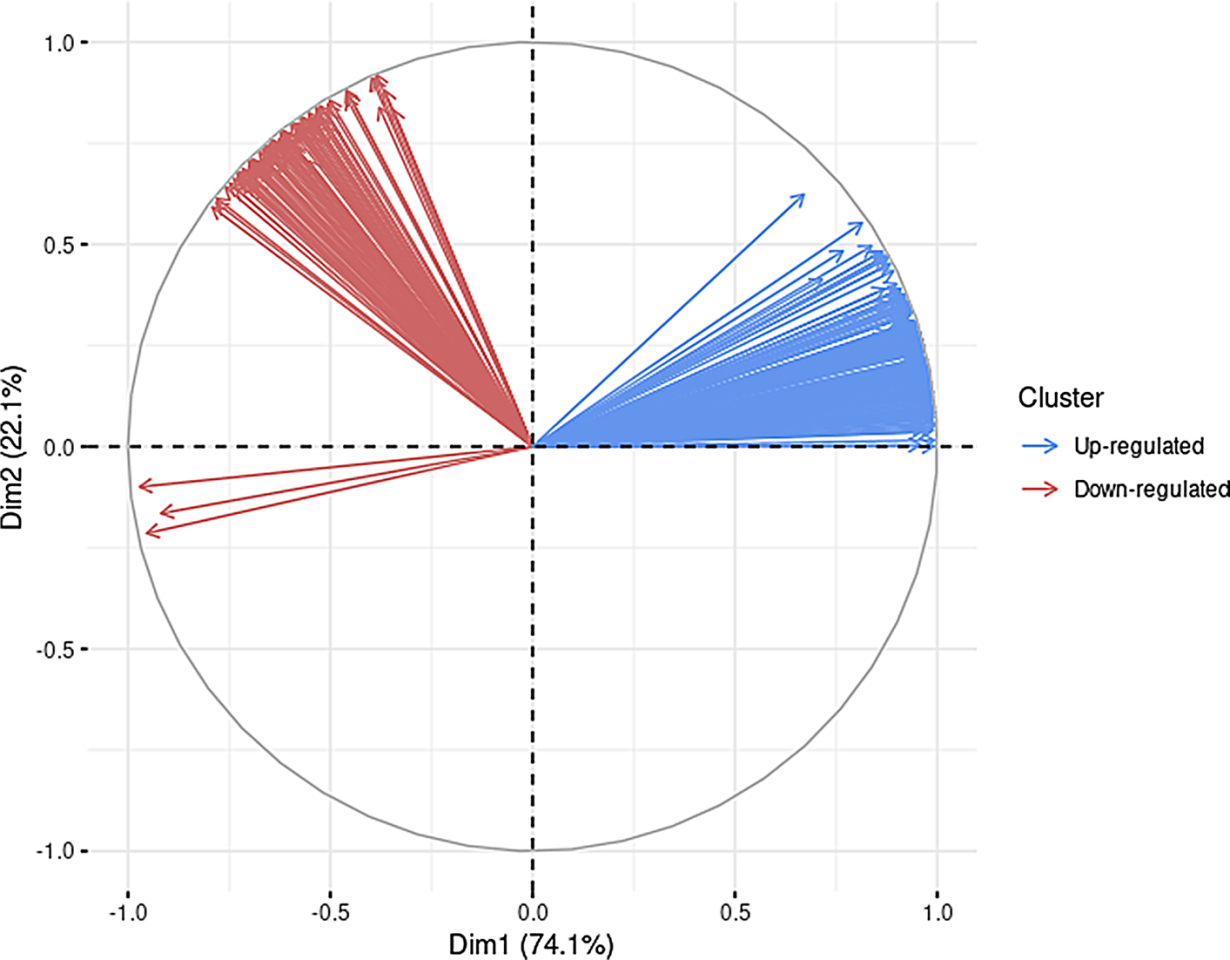
PCA graphical representation of the variables of the model using significant transcripts after the ANOVA-Dunnett test and power analysis filtering. Principal Components (PC) 1 and 2 represent 96.2% of the variability of the data and oppose two clear clusters in relation to the Dim1 axis, that explains most variability (74.1%). The positive cluster in relation to this axis corresponds with the up-regulated cluster (Fig. 4a) while the transcripts included in the negative axis according to the Dim1 axis correspond to the ones that decrease their expression after death (Fig. 4b).

**Figure 4:**
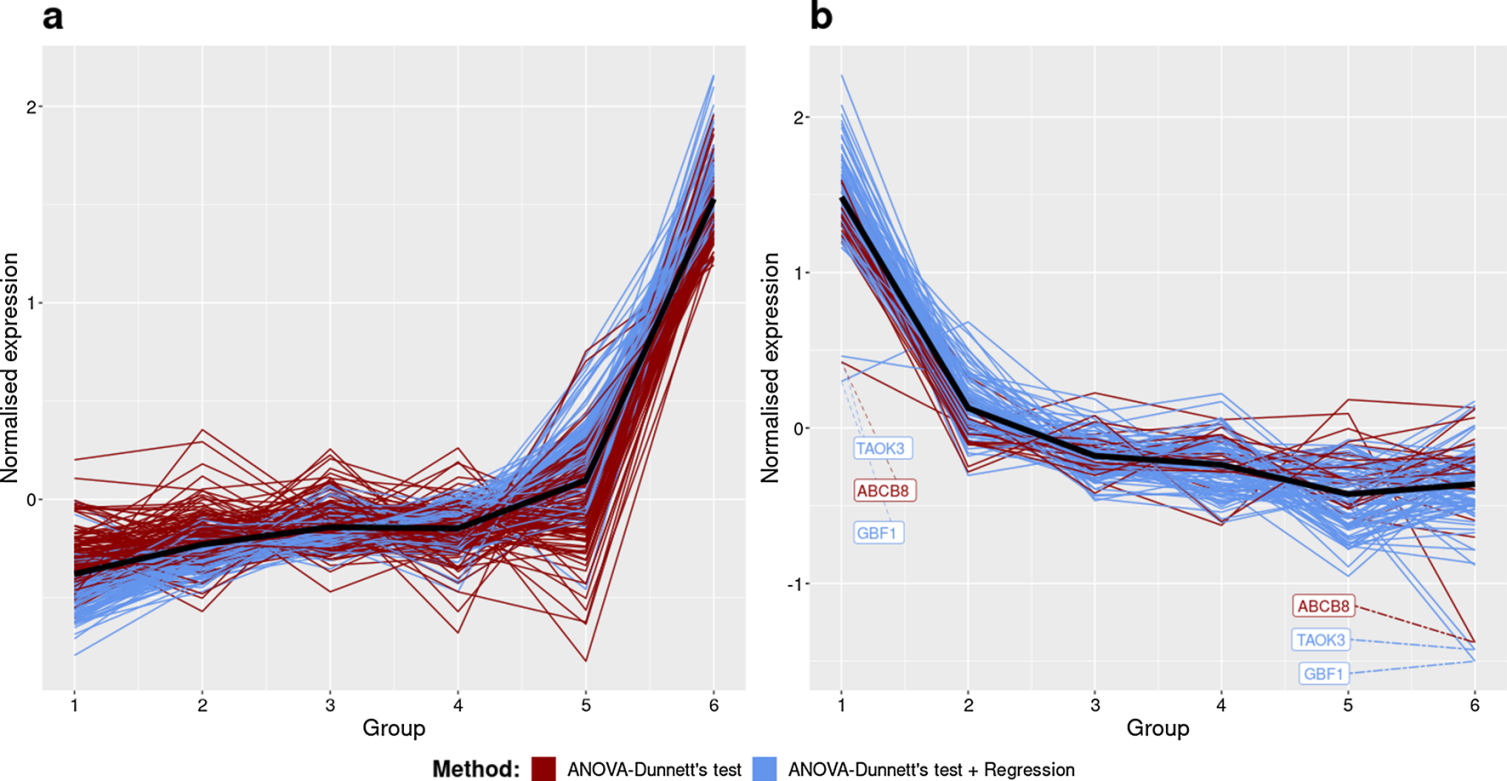
Mean of the expression value of the transcripts included in (**a**) up-regulated cluster and (**b**) down-regulated cluster shown in Fig. 3 after the ANOVA-Dunnett’s test and power filtering. For each transcript, the mean expression in every bin is represented. Transcripts included in the first cluster (227 transcripts) show an increase in expression after death while transcripts included in the second cluster (108 transcripts) show a decrease in expression over time. The black line represents the mean of all transcripts per bin. Colors show whether those transcripts were also identified using linear regression (blue) or not (red). Only blue lines represent the input for downstream analyses.

**Figure 5:**
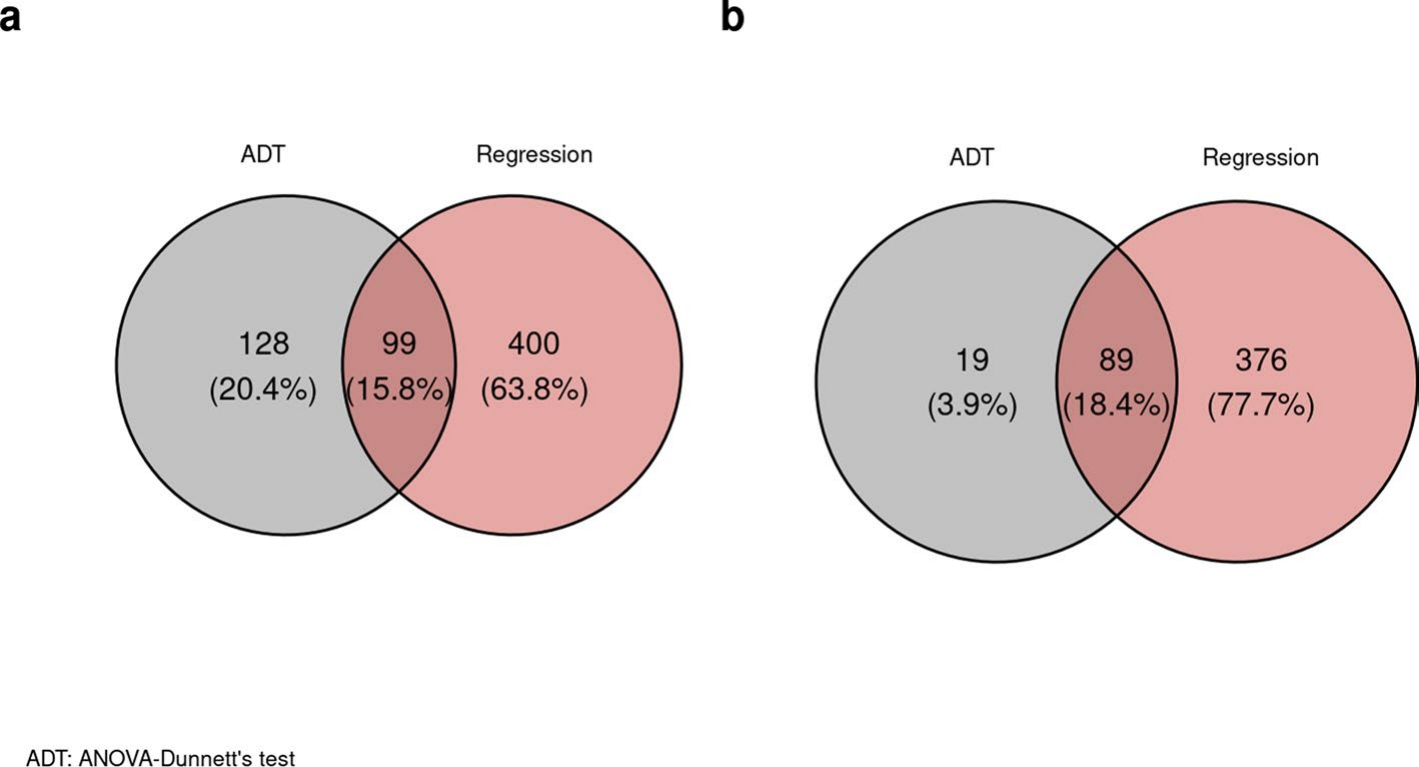
Venn’s diagram for significant transcripts after power filtering (**a**) Up-regulated transcripts both for ANOVA-Dunnett (n = 227) and linear regression analysis (n = 499). (**b**) Down-regulated transcripts also identified with ANOVA-Dunnett (n = 108) and linear regression analysis (n = 465). Only common genes between both methods (n = 99 and n = 89) were used in downstream analyses.

### Identification of PM gene expression patterns

Clustering of the PM gene expression data was suggested by the Hopkins statistic (*H* = 0.60). ANOVA with a Dunnett post-hoc test between the early (2 h 21 min–8 h 00 min) and the late PMI bin (PMI 34 h 00 min–40 h 30 min) identified 227 up-regulated and 108 down-regulated transcripts after power analysis (see Supplementary spreadsheet 1). PCA of the these transcripts showed two separated and clearly opposed clusters while 3 transcripts clustered together but were separated from the two main groups (Fig. 3). The mean expression of those two clusters confirmed our initial hypothesis of up- and down-regulated transcripts after death (Fig. 4a,b). Moreover, the 3 independent arrows (corresponding to genes *GBF1*, *ABCB8* and *TAOK3*) also showed decreased expression after death. These transcripts were therefore included in the down-regulated cluster of transcripts for downstream analysis. On the other hand, linear regression analysis identified 499 up-regulated and 465 down-regulated transcripts after power filtering (see Supplementary spreadsheet 2) . Finally, 99 up-regulated transcripts (Fig. 5a) and 89 down-regulated transcripts (Fig. 5b) were identified with both methods as they showed a continuous change in expression (Supplementary Tables S1 and S2) with a significant difference between the early (2 h 21 min–8 h 00 min) and late PMI bins (PMI 34 h 00 min–40 h 30 min). Supplementary Figures S2a and S2b show the patterns of the mean expression of the transcripts included in the up-regulated and down-regulated clusters, respectively while examples of gene expression patterns of the top three up- and down-regulated patterns are depicted in Supplementary Figures S3A and S3B.

### GO enrichment analysis

**Figure 6:**
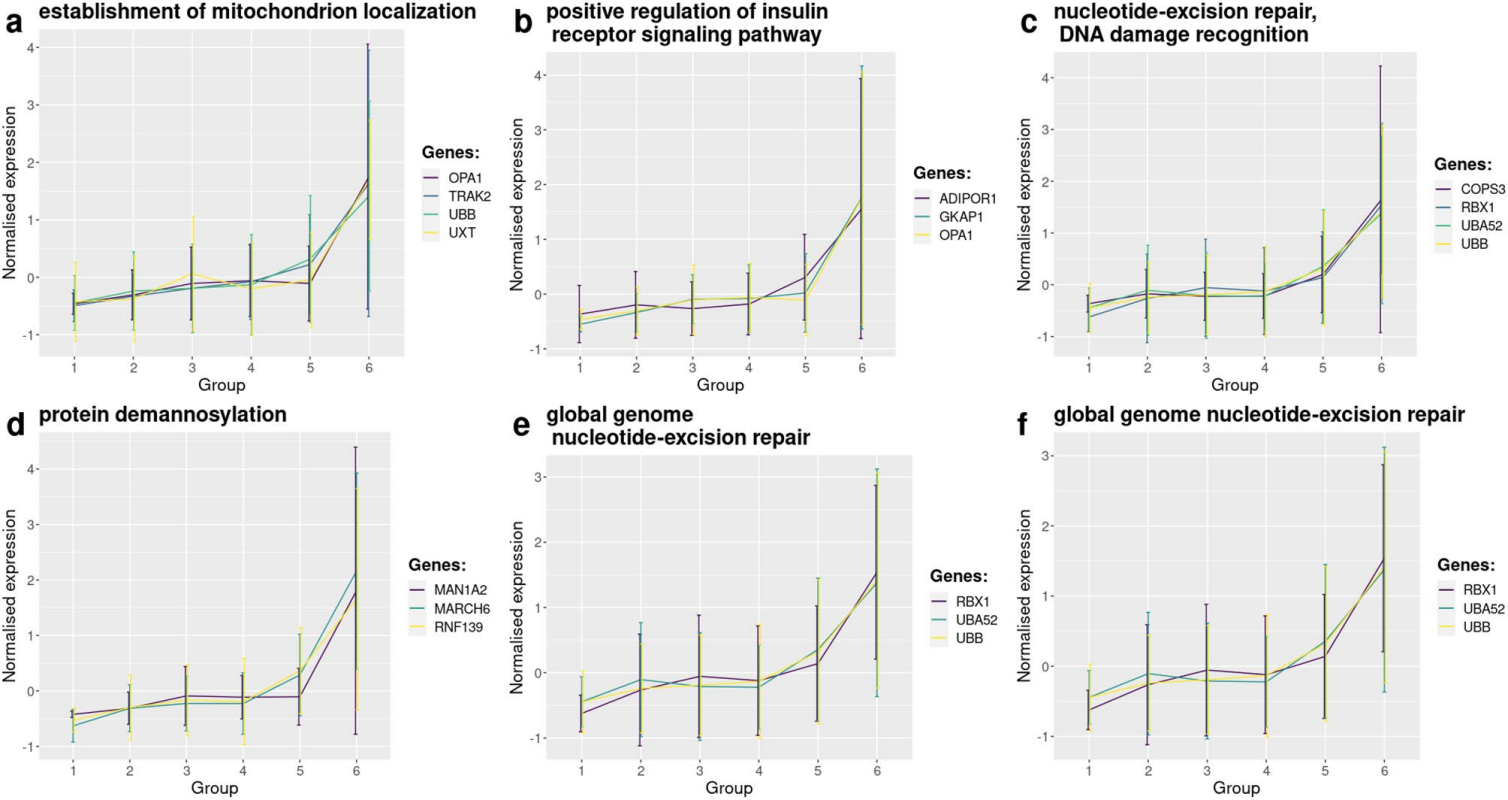
Top 6 processes enriched by genes found to be significantly expressed in the up-regulated cluster between the early (2 h 21 min–8 h 00 min) and the late PMI bin (PMI 34 h 00 min–40 h 30 min). For each process, the mean expression, per bin, of every gene involved is represented.

GO enrichment analyses were performed for both significantly up-regulated and down-regulated transcripts. The highest enriched processes within the up-regulated transcripts were the establishment of mitochondrion localization, the positive regulation of insulin receptor signaling and nucleotide excision repair (Table 1). The significant genes included in each of the top enriched processes are listed in Table 2. A representation of the expression of these genes can be seen in Fig. 6. On the other hand, processes involved in the death-inducing signaling complex assembly, the positive regulation of macrophage differentiation, the toll-like receptor 3 signaling pathway and the regulation of necrotic/necroptotic cell death were most enriched among the down-regulated genes (Table 3). These enriched GO terms were represented through the same 3 genes (*CASP8*, *RIPK1*, and *FADD*).

**Table 1:** Top enriched pathways, ordered by decreasing odds ratio, with their corresponding GO term identifier and pathway. The input data were the differentially expressed transcripts in the up-regulated cluster according to the ANOVA-Dunnett’s test and regression (99 genes). The expected count and the actual number of genes of the input that are included in a GO term are those used in the statistical analysis, in order to calculate the *p*-value. Size is the total number of genes included in that particular GO term.

GOBPID	p-value	FDR	Odds ratio	Exp count	Count	Size	Term
GO:0051654	8.4889e−06	4.3294e−04	40.97	0.1382	4	15	Establishment of mitochondrion localization
GO:0046628	3.1732e−04	4.0459e−03	27.82	0.1382	3	15	Positive regulation of insulin receptor signaling pathway
GO:0000715	5.2057e−05	1.3274e−03	23.70	0.2120	4	23	Nucleotide-excision repair, DNA damage recognition
GO:0036507	5.5783e−04	4.6914e−03	22.25	0.1659	3	18	Protein demannosylation
GO:0000717	7.6902e−04	4.6914e−03	19.63	0.1843	3	20	Nucleotide-excision repair, DNA duplex unwinding
GO:0070911	1.1711e−03	5.9416e−03	16.68	0.2120	3	23	Global genome nucleotide-excision repair

**Table 2:** Top 6 enriched processes found with the GO enrichment analysis using up-regulated transcripts in the late PMI bin (PMI 34 h 00 min–40 h 30 min) compared to the early (2 h 21 min–8 h 00 min) PMI bin. Term ID correspond to the pathway ID and the genes are the ones involved in that specific biological process, also found in the cluster.

Term ID	Genes
GO:0051654	OPA1, TRAK2, UBB, UXT
GO:0046628	OPA1, ADIPOR1, GKAP1
GO:0000715	UBA52, UBB, COPS3, RBX1
GO:0036507	MARCHF6, MAN1A2, RNF139
GO:0000717	UBA52, UBB, RBX1
GO:0070911	UBA52, UBB, RBX1

### PMI prediction modelling

**Figure 7:**
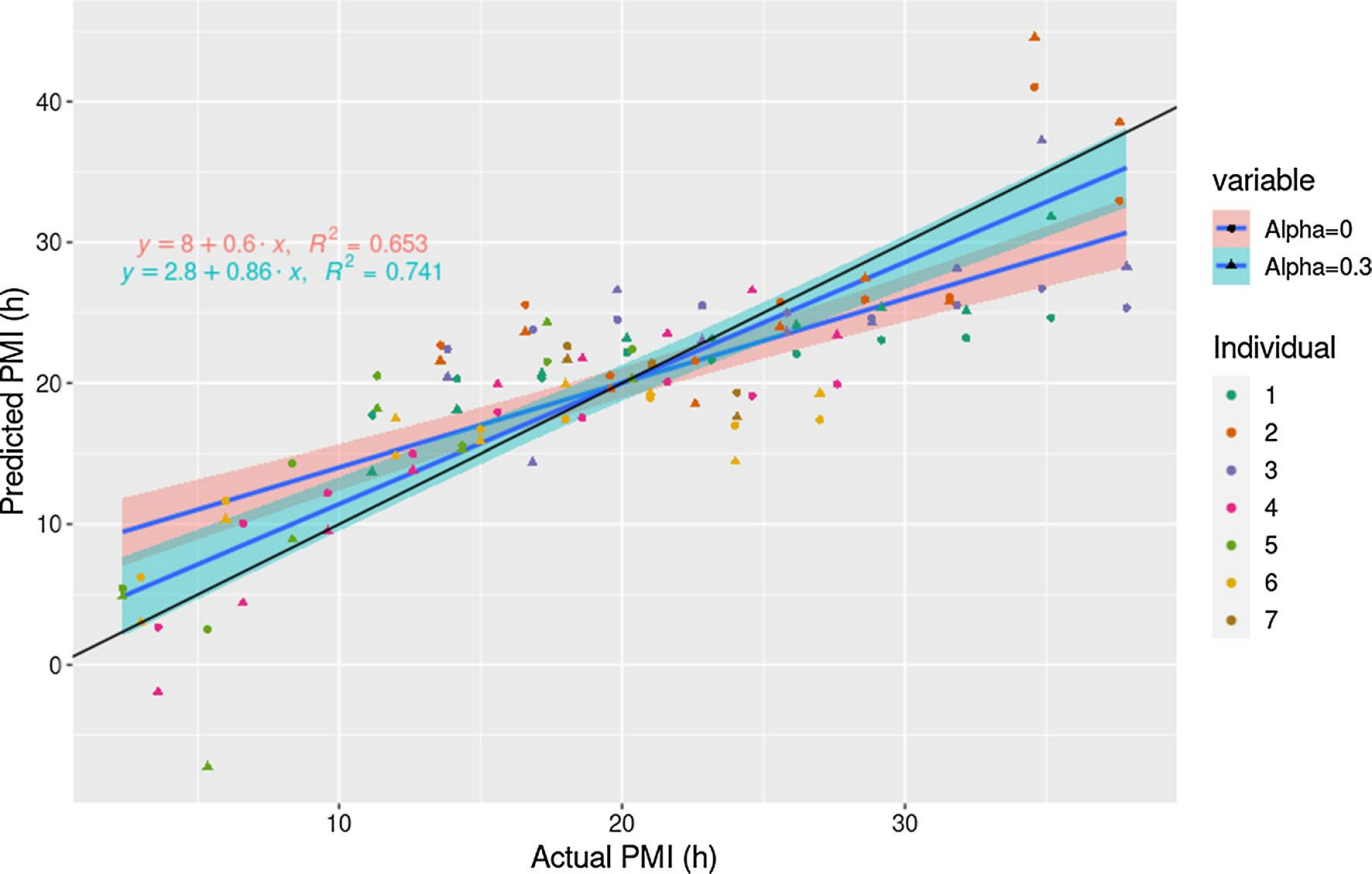
Actual vs. predicted PMI values for alpha 0 (using all genes, red color) and 0.3 (lowest RMSE obtained, blue color) in the glmnet 10-repeat-5-fold CV test. The linear regression of both alpha values is also shown, together with the formula and the R^2^ value.

The third objective of this paper was to develop a PMI prediction model from the transcript expression data obtained using the current longitudinal study. The mean RMSE obtained from a 10-repeat 5-fold CV for all alpha values tested ranged from 4.75 to 5.56 h (Table 4). The number of transcripts used in the models ranged from 16 to 189 (all the transcripts identified in common between ANOVA and regression plus the intercept). The lowest RMSE of 4.75 h was obtained using 30 genes (alpha = 0.3) (Table 5). The mean of their coefficient values ranged from − 0.75 to 1.11 and those transcripts with a positive coefficient were found to be included in the up-regulated cluster and those with a negative slope were included in the down-regulated cluster. Predicted vs. expected PMI values were compared between models using 188 vs. 30 transcripts and showed an overall overestimation of lower PMI values and an underestimation of late PMIs after 20 h PM (Fig. 7, Table 6).

**Table 3:** Top enriched pathways, ordered by decreasing odds ratio, with their corresponding GO term identifier and pathway. The input data were the differentially expressed transcripts in the down-regulated cluster according to the ANOVA-Dunnett test and regression (89 genes). The expected count and the actual number of genes of the input that are included in a GO term are those used in the statistical analysis, in order to calculate the *p*-value. Size is the total number of genes included in that particular GO term.

GOBPID	p-value	FDR	Odds ratio	Exp count	Count	Size	Term
GO:0071550	5.2776e−05	1.6663e−03	58.56	0.0790	3	9	Death-inducing signaling complex assembly
GO:0045651	1.0236e−04	1.9191e−03	43.91	0.0966	3	11	Positive regulation of macrophage differentiation
GO:0060546	1.3561e−04	1.9191e−03	39.03	0.1054	3	12	Negative regulation of necroptotic process
GO:0060547	1.3561e−04	1.9191e−03	39.03	0.1054	3	12	Negative regulation of necrotic cell death
GO:0034138	3.3655e−04	2.8943e−03	27.01	0.1405	3	16	Toll-like receptor 3 signaling pathway
GO:0062098	3.3655e−04	2.8943e−03	27.01	0.1405	3	16	Regulation of programmed necrotic cell death

**Table 4:** Summary of the results of the 10-repeat 5-fold predictions with glmnet. The results represent the mean of 10 repetitions. alpha represents the elastic net factor, RMSE is the mean RMSE found in each of the 10 repetitions, SD is the mean SD found in each repetition, Num. factors is the number of transcripts (plus the intercept) used to calculate each model and the lambda is also a mean between the 10 repetitions.

Alpha	RMSE	SD	Num. factors	Minimum lambda
0.00	5.30	0.11	189	63.57
0.10	5.14	0.28	65	4.48
0.20	5.35	0.27	38	1.70
0.30	4.75	0.24	31	1.98
0.40	5.00	0.14	32	0.61
0.50	5.56	0.40	22	1.43
0.60	4.92	0.24	19	1.43
0.70	4.90	0.25	18	0.44
0.80	5.21	0.18	17	0.74
0.90	5.22	0.26	16	0.87
1.00	5.28	0.29	16	0.75

Num. factors = genes used in the model + intercept.

**Table 5:** Coefficients of the contributing transcripts when the prediction model was performed with alpha = 0.3. Shown are the 30 transcripts that were present in all the repetitions and the coefficient is the mean of the 10 repetitions. All transcripts were found as significant with the linear regression method, after power. Transcripts with a positive slope were included in the up-regulated cluster and transcripts with a negative slope were included in the down-regulated cluster.

Gene ID	Gene name	Coefficients	Function
(Intercept)		20.01	
ENSG00000253982	AC100810.1	1.11	LncRNA^65^
ENSG00000155090	KLF10	1.06	PCG: Plays a role in the regulation of the circadian clock^66^
ENSG00000145495	MARCH6	0.84	E3 ubiquitin-protein ligase that promotes ’Lys-48’-linked ubiquitination of target proteins, leading to their proteasomal degradation^66^
ENSG00000186063	AIDA	0.68	PCG: Acts as a ventralizing factor during embryogenesis^66^
ENSG00000256053	APOPT1	0.23	PCG: Initiates apoptosis by triggering release of cytochrome *c*^67^
ENSG00000187479	C11orf96	0.16	PCG^67^
ENSG00000145354	CISD2	0.15	PCG: Regulator of autophagy that contributes to antagonize BECN1-mediated cellular autophagy at the endoplasmic reticulum^66^
ENSG00000038210	PI4K2B	0.09	Type II PI4 kinase involved in early T Cell activation^67^
ENSG00000167528	ZNF641	− 0.01	Zinc Finger, transcriptional activator. Activates transcriptional activities of SRE and AP-1^66^
ENSG00000272941	AC083862.2	− 0.03	LnCRNA^65^
ENSG00000165609	NUDT5	− 0.06	Enzyme that can either act as an ADP-sugar pyrophosphatase in absence of diphosphate or catalyze the synthesis of ATP in presence of diphosphate^66^
ENSG00000131043	AAR2	− 0.09	Homolog of the yeast A1-alpha2 repressin protein that is involved in mRNA splicing^67^
ENSG00000107862	GBF1	− 0.10	PCG involved in vesicular trafficking by activating ADP ribosylation factor 1^67^
ENSG00000168282	MGAT2	− 0.10	PCG: Plays an essential role in protein N-glycosylation^66^
ENSG00000140598	EFL1	− 0.13	PCG: Transcription factor that activates the LYN and BLK promoters^66^
ENSG00000118217	ATF6	− 0.16	PCG: Activates target genes for the unfolded protein response (UPR) during endoplasmic reticulum (ER) stress^66^
ENSG00000171049	FPR2	− 0.17	PCG: Neutrophil chemotactic factor^66^
ENSG00000169508	GPR183	− 0.19	PCG: chemotactic receptor for B-cells, T-cells, splenic dendritic cells, monocytes/macrophages and astrocyte^66^
ENSG00000104228	TRIM35	− 0.20	PCG: Participates in multiple biological processes including cell death, glucose metabolism, and in particular, the innate immune response^66^
ENSG00000166927	MS4A7	− 0.22	PCG: May be involved in signal transduction^66^
ENSG00000095370	SH2D3C	− 0.23	PCG: Acts as an adapter protein that mediates cell signaling pathways involved in cellular functions such as cell adhesion and migration, tissue organization, and the regulation of the immune response^66^
ENSG00000163823	CCR1	− 0.29	PCG: Transduces a signal by increasing the intracellular calcium ions level^66^
ENSG00000133313	CNDP2	− 0.34	APCG: Acts as a functional tumor suppressor in gastric cancer via activation of the mitogen-activated protein kinase (MAPK) pathway^66^
ENSG00000110777	POU2AF1	− 0.34	PCG: It is essential for the response of B-cells to antigens and required for the formation of germinal centers^66^
ENSG00000133561	GIMAP6	− 0.41	PCG: Member of the GTPases of immunity-associated proteins family^67^
ENSG00000101347	SAMHD1	− 0.48	PCG: Acts both as a host restriction factor involved in defense response to virus and as a regulator of DNA end resection at stalled replication forks^66^
ENSG00000168040	FADD	− 0.57	PCG: Apoptotic adaptor molecule that recruits caspase-8 or caspase-10 to the activated Fas (CD95) or TNFR-1 receptors^66^
ENSG00000185009	AP3M1	− 0.67	PCG: Part of the AP-3 complex required to target cargo’s into vesicles assembled at cell bodies for delivery into neurites and nerve terminals^66^
ENSG00000171860	C3AR1	− 0.67	PCG: Receptor that stimulates chemotaxis, granule enzyme release and superoxide anion production^66^
ENSG00000198130	HIBCH	− 0.75	PCG: Saline catabolite with high activity toward isobutyryl-CoA^66^

LncRNA: long non-coding RNA; PCG: Protein coding gene;

**Table 6:** Mean of the 10-repeat prediction values for alpha = 0 and alpha = 0.3. For each sample, the known PMI is shown and then the mean PMI predicted in each of the 10 repetitions with alpha = 0 and alpha = 0.3.

	PMI	Alpha = 0	Alpha = 0.3
DEG1_0	11.17	17.72	13.72
DEG1_3	14.17	20.28	18.12
DEG1_6	17.17	20.37	20.68
DEG1_9	20.17	22.18	23.18
DEG1_12	23.17	21.65	23.14
DEG1_15	26.17	22.10	24.12
DEG1_18	29.17	23.05	25.35
DEG1_21	32.17	23.22	25.12
DEG1_24	35.17	24.64	31.85
DEG2_0	13.58	22.69	21.60
DEG2_3	16.58	25.57	23.66
DEG2_6	19.58	20.51	19.56
DEG2_9	22.58	21.56	18.52
DEG2_12	25.58	25.76	24.02
DEG2_15	28.58	25.93	27.44
DEG2_18	31.58	26.09	25.84
DEG2_21	34.58	41.04	44.58
DEG2_24	37.58	32.97	38.56
DEG3_0	13.83	22.40	20.42
DEG3_3	16.83	23.78	14.38
DEG3_6	19.83	24.53	26.60
DEG3_9	22.83	25.49	23.01
DEG3_12	25.83	25.01	23.61
DEG3_15	28.83	24.58	24.33
DEG3_18	31.83	25.54	28.16
DEG3_21	34.83	26.73	37.22
DEG3_24	37.83	25.35	28.29
DEG4_0	3.60	2.66	− 1.91
DEG4_3	6.60	10.06	4.40
DEG4_6	9.60	12.19	9.50
DEG4_9	12.60	14.97	13.80
DEG4_12	15.60	17.95	19.90
DEG4_15	18.60	17.54	21.73
DEG4_18	21.60	20.09	23.51
DEG4_21	24.60	19.09	26.58
DEG4_24	27.60	19.92	23.39
DEG5_0	2.35	5.44	4.85
DEG5_3	5.35	2.54	− 7.25
DEG5_6	8.35	14.31	8.90
DEG5_9	11.35	20.51	18.19
DEG5_12	14.35	15.57	15.33
DEG5_15	17.35	21.54	24.30
DEG5_18	20.35	22.39	20.28
DEG6_0	3.00	6.22	3.00
DEG6_3	6.00	11.65	10.36
DEG6_9	12.00	14.85	17.47
DEG6_12	15.00	16.70	15.86
DEG6_15	18.00	17.40	19.89
DEG6_18	21.00	18.92	19.31
DEG6_21	24.00	17.00	14.44
DEG6_24	27.00	17.39	19.28
DEG7_0	18.07	22.61	21.69
DEG7_3	21.07	21.42	21.28
DEG7_6	24.07	19.32	17.58

## Discussion

### PM gene expression patterns

Our data are the first (to our knowledge) to report on a longitudinal human thanatotranscriptomic study. PM human blood samples were collected from individuals with a known ToD over a period of maximum 38 h after death with a regular time interval at constant room temperature. The gene expression data was produced by 3$'$ end mRNA-seq using a standard sequencing depth (5 million reads per sample) after globin RNA depletion. This strategy aimed to detect more highly expressed transcripts even when the 5$'$ end of the transcripts is not available any more due to degradation. Because the sequencing was done towards the 3$'$ end of the mRNA shorter reads were detected more efficiently, and the number of reads aligned per transcript did not depend on the original transcript length. Furthermore, the globins eclipsing effect was avoided by filtering in the library preparation steps. As decomposition is known to start almost immediately after death as cells initiate the process of autolysis for self-destruction through enzymatic digestion^26^ , rapid changes in gene expression can be expected due to RNA degradation. Transcriptome sequencing of the PM blood samples collected in the current study did indeed identify genes that showed a continuous decrease after death. Interestingly, genes were also identified that showed a clear up-regulated pattern. Similar patterns have been observed previously in several animal^13,27^ and human tissues^9,12,15,16^ but this is the first time these patterns were demonstrated in a longitudinal human study.

Three individual transcripts (*ABCB8*, *GBF1*, *TAOK3*) clustered together but were separated from the two main clusters in the PCA plot (Fig. 3). These three transcripts showed decreased expression over time and were therefore included in the down-regulated cluster for downstream analysis. We hypothesize that these three transcripts cluster separately as the decreasing pattern is different from the other genes (Fig. 4). The *ABCB8* gene encodes an ATP-binding cassette protein and is essential for iron homeostasis in mitochondria^28^, possibly by acting as an iron exporter^29^. The *GBF1* gene produces a guanine nucleotide exchange factor and is localised in the Golgi apparatus. It regulates vesicular trafficking and mitochondrial positioning in cells in a microtubule-dependent manner^30^. *TAOK3* encodes for a serine/threonine kinase and was demonstrated to play a role in insulin resistance^31^. The different clusters identified through PCA suggest that transcripts might have different susceptibilities to degradation or are actively regulated in the dying cell. In order to find out which hypothesis would be more likely, a GO enrichment analysis was performed.

### GO enrichment analysis

Gene ontology enrichment analysis was performed in order to shed light on the processes that are up- and down-regulated after death. Up-regulated genes comprised several processes involved in the regeneration of cells. The nucleotide excision repair (NER) pathway was part in 3 out of the top 6 enriched pathways and is essential in repairing damaged DNA at single nucleotide level. An early rise in postmortem oxidative stress was previously observed in PM muscle tissue obtained from adult male Wistar rats^27^. Oxidative stress as measured through the levels of intracellular reactive oxygen species (ROS) and reactive nitrogen species (RNS) are well known to induce DNA damage via oxidation and DNA strand breaks^32^. As a response to the increase in cellular damage the cell might therefore initiate the NER pathway to reduce or minimize the damage done. A second highly enriched GO term was protein demannosylation. While mannosidic glycoepitopes are vital for maintaining proteostasis^33^, aberrant glycosylation patterns have been hypothesised to be part of stress-induced or danger signals and thereby reflect the cell’s phenotypic status to incite an immunological response^34^. Finally, the process of mitochondrial localization has a role in the maintenance of DNA within mitochondria by binding the nucleoids (containing the mitochondrial DNA) to the inner membrane^35^. As an example, optic atrophy protein 1 (*OPA1*) has been demonstrated to have a role in mitochondrial inner membrane fusion and knockdown of *OPA1* resulted in mitochondrial fragmentation, potential dissipation, and cristae disorganization, all of which were associated with cytochrome-*c* release located at the inner membrane and caspase-dependent apoptotic cell death^36–38^.

Multiple processes involved in cell death were enriched among the down-regulated genes but were represented through 3 common genes: *CASP8*, *RIPK1* and *FADD* (Table 3). Cell death in mammals can be activated through two distinct pathways: (i) the intrinsic or mitochondrial pathway and (ii) the extrinsic pathway through cell death receptors^39^. *Caspase-8* (*CASP8*) is involved in programmed cell death as a proapoptotic protease. The Death Effector Domain of FADD, a signal transducer downstream of cell death receptor *CD95* (also called *Fas*), binds to the N-terminal prodomain of caspase-8 resulting in cell death^40,41^. Both *FADD* and *CASP8* are essential during murine embryogenesis while cells in mice deficient in *FADD* or *CASP8* are resistant to death receptor-induced apoptosis^42^. *CASP8* has also been shown to interact with *RIPK1* to induce apoptosis^43–45^. Whereas apoptosis is mediated by the caspase-pathway , necroptosis is a form of regulated necrotic cell death that can be activated under apoptosis-deficient conditions^46^. *RIPK1* can play a role in both processes depending on the cell type and context^47,48^. *CASP8* and *RIPK2*, but not *RIPK1*, was also shown to be down-regulated by Javan and colleagues^16^ in PM liver samples. In the same study *FADD*, however, was up-regulated.

Gallego Romero and colleagues suggested that actively mediated degradation of transcripts may occur during necrosis; namely, that degradation of RNA in a dying tissue may not be a completely random process and that the relative importance of stochastic degradation may increase such that at later time-points degradation becomes increasingly uncoupled from biological function^49^. Ferreira and colleagues stated that their pathway analysis suggested active regulation^12^, whereas Pozhitkov and colleagues considered that their data suggested a step-wise shutdown after death^13^. Pozhitkov and colleagues detected an increase of developmental genes^13^ while Ferreira and colleagues detected the deactivation of the immune system and an increase of processes related to blood coagulation and responses to stress^12^.

Taken together, our results suggest that soon after death cells actively switch to survival mode through the activation of DNA repair pathways and suppress apoptosis and necroptosis-related pathways and that mitochondria take a lead role in these PM processes. Further studies with larger data sets might reveal additional PM gene expression patterns besides the ones uncovered in the current study. These patterns might also shed more light on RNA degradation rates of specific transcripts.

### PMI prediction

The third objective of this report was to develop a prediction model based on PM gene expression data to estimate the PMI. We were able to develop a model with an RMSE of 4.75 h using a small number of transcripts (n = 30). Figure 7 shows that there is an overestimation of PMI during the first hours after death, while after 20 h PM the predictions tend to underestimate the variable. Other studies have previously reported the use of RNA-seq data in order to predict PMI from gene expression after death. Ferreira and colleagues obtained an R^2^ of 0.77 between observed and expected PMI when they used the information of all the tissues available in the GTEx project^12^. They do not show a correlation parameter only for blood, however, we do consider that obtaining an R^2^ = 0.741 (when alpha = 0.3), despite the limited number of samples, supports the hypothesis that this type of data holds great promise for PMI prediction modeling. Whether the model might be valid for younger individuals is currently unknown, but as specific mRNA markers are associated with age and can be thus used to estimate age, this would be an interesting topic of future research^50^.

### Limitations of the study

The experimental design used in this study has some critical advantages and disadvantages that need to be taken into account when interpreting the results of the study. Due to the restrictions of a known ToD only a limited number of participants were able to be recruited in the study. In addition, the PM blood coagulation process starts as soon as the blood flow ceases and reaches the dependent parts of the body after 6–8 h^51^. This process is dependent on many factors, such as the amount of available blood in the veins, and has hampered the collection of samples in individual 7 where only 3 PM blood samples were able to be collected. For the same reason the maximum incubation time of 36 h was chosen as blood is rarely available for collection through the needle aspiration collection process after 36 h.

In our study bodies were kept at room temperature for up to 38 h PM. It is currently unknown what the effect is of either the endogenous or exogenous microbiome on the thanatotranscriptome and therefore also on the prediction accuracy of the PMI model. Evidence for a healthy blood-microbiome is still sparse^52^ but translocation of intestinal bacteria to extra-intestinal locations such as cardiac blood has been observed in sacrificed mice after 5 min^53^. Future research could investigate the effect of migrating microorganisms and their effect on the thanatotranscriptome of the host while taking care to avoid contamination from other locations than the intended sampling site by employing validated decontamination procedures.

Because the study was carried out under a controlled environment with a high age group and limited PMI, the effect of a changing environment, age and late PMI (> 38 h) on gene expression patterns could not be evaluated and therefore restrict the suitability of the model for real-life cases.

## Conclusion

This study reports on a novel approach to increase our understanding of gene transcription after death using a longitudinal experimental design whereby multiple human PM blood samples were collected at regular time intervals from the same individuals with a known ToD. We demonstrate that at least two main distinguishing types of behavior of up- and down-regulated transcripts are present up to 38 h after death. Moreover, we provide evidence of actively regulated processes involved in the regeneration of the cell through DNA damage repair and the suppression of apoptotic and necroptotic pathways. Finally, a PMI prediction model with an accuracy of 4.75 h up to 38 h PM was developed based on 30 RNA transcripts.

## Materials and methods

### Sample collection

PM blood samples were collected from deceased individuals with a known time of death who donated their body to science. All individuals had died within the hospital (University Hospitals Leuven, Leuven, Belgium) and were immediately transferred to a private room upon arrival at the morgue of the hospital. From the ToD until the end of the entire sampling procedure the bodies were kept at room temperature (${18}^\circ$) . The cause of death of the individuals were heart failure, septic shock, staphylococcus infection, intracranial bleeding, septic shock and euthanasia for patients 1 to 7 respectively. No specific mRNA markers could be linked to either cause of death and therefore no transcripts were excluded on the basis of cause of death. Blood samples were taken during a time frame of 24 h starting upon arrival and with a 3 h interval (i.e. 7 blood samples per individual if possible). PMI was calculated as Sampling time (h) − Time of death (h). Blood was alternately collected from the *vena femoralis* (left and right) and *vena subclavian* (left and right). If blood collection failed at one location, sampling continued from another location until 2.5 mL was collected in a PAXgene Blood RNA Tube (IVD). Ethical approval for this study was obtained from the Ethical Commission of the University Hospitals Leuven (case number S58486) and informed consent was obtained from the participants before donation. The study was performed in accordance with the relevant internal guidelines as well as the Declaration of Helsinki.

### RNA preparation and sequencing

Blood samples were incubated at room temperature for 5 h after which they were stored at ${-20}^\circ$ for 24 h and transferred to ${-\,80}^\circ$ as described by the manual. RNA extraction was performed using the PAXgene Blood RNA Kit (IVD) according to manufacturer’s instructions. RNA quantification was performed using NanoDrop spectrophotometry (Thermo Fisher Scientific) and RNA quality was assessed on the Bioanalyzer 2100 using the RNA 6000 Nano Kit (Agilent). RNA samples were stored in ${-\,80}^\circ$ until cDNA library preparation. cDNA libraries were created using the BlueBee mRNA-Seq Library Prep Kit FWD for Illumina (Lexogen) including the Globin Block module to remove the majority of the globin mRNA transcripts. 100 ng RNA was used during first strand cDNA synthesis. Library amplification was performed using 16 PCR cycles. cDNA pools were quantified using the Qubit dsDNA HS assay (Thermo Fisher Scientific). Sizing of the pools was performed on the BioAnalyzer 2100 using the High Sensitivity DNA assay (Agilent). Final cDNA libraries were pooled and sequenced on the Illumina NextSeq platform (single read, high output, 75 bp). The bioinformatics pipeline was performed on the BlueBee platform. This platform has a specific pipeline to process reads originating from Lexogen’s QuantSeq 3$'$ mRNA Library Prep Kit and includes quality control steps, trimming and read alignment to the human reference genome (GRCh38) using STAR alignment^54^. Number of reads per sample can be seen in Supplementary Figure S1. Raw read counts were normalised in BlueBee using the DeSeq2 pipeline using the median of ratio’s method^55^. After standardisation, transcripts with zero reads in 6 samples or more were filtered out to avoid random counts. Out of a total of 60.199 transcripts, 10.635 remained and were used as input for the downstream analyses. All data was standardised using z-scores.

### Identification of PMI-associated genes

*All statistical analyses were performed in R (version 3.6.3)* ^56^

An overview of the study pipeline is provided in Fig. 1. A total of 54 samples from 7 individuals were collected and used in the statistical analyses.

#### Clustering assessment

Two methods were performed in order to evaluate if the data was randomly or uniformly distributed, rather than clustered: mathematically, with the Hopkins statistic, and visually, with a PCA analysis in relation to PMI using Factoextra (v. 1.0.5)^57^ and FactoMineR (v. 1.42)^58^.

#### ANOVA and Dunnett’s test

All samples were ordered by increasing PMI and then grouped in bins. Analysis of Variance (ANOVA) was performed on the means of all transcript expression values between each bin. With the package multcomp (v. 1.4.10)^59^ a Dunnett’s post-hoc test was used to filter out results which were at least significant between the early PMI and late PMI bins. All PMI bins were therefore included in the ANOVA and posterior Dunnett’s test. The significant transcripts were divided into clusters according to the previous PCA results. Additionally, a Supplementary spreadsheet 3 was included, with the ANOVA-Dunnett’s test results between increasing bins (e.g.: 1 vs. 2–6, 1–2 vs. 3–6, 1–3 vs. 4–6, 1–4 vs. 5–6 and 1–5 vs. 6). The number of significant genes for each comparison were 1247, 1364, 1142, 2155 and 1646, respectively.

#### Regression analysis

Regression was also used to analyze the PMI correlation with gene transcription. For each transcript, the linear model parameters (the slope and a constant) were estimated from the samples, and a hypothesis test determined whether the slope was significantly different from zero. The *p*-value was calculated with a built-in-function of the *stats package*^56^ and those transcripts with a value lower than 0.05 were considered significant, and then assigned to a cluster depending on the sign of the slope.

#### Power analysis

Power analysis was performed twice: a first time with the ANOVA-Dunnett’s significant results, and a second time for the regression analysis significant results. In both cases the significant results were filtered again, and only those with power greater than 0.8 were included in the pathway analysis.

For the first power test, the package used was pwr (v. 1.2.2)^60^. The parameters needed were the number of groups (*k* = 2, the control bin and the last bin), the samples size (the mean of the number of samples included in the control group and in the bin number 6), and the effect size. Because the *SD* was 1 for all the transcripts, the effect size in this analysis was the mean of the expression value in the last bin minus the mean of the expression value in the control group. The significance, alpha, was set to be the standard 0.05. For the power calculation over the regression analysis results, the same significance was chosen. The package used in this case was WebPower (v. 0.5.2)^61^. The other parameters were the sample size (*n*=54), the number of predictors (*p*=1, referring to PMI) and the effect size *f* was calculated for each transcript as the effect size proposed in the manual of the package^62^ (i.e.: $f^2 = R^2/ (1-R^2)$).

### GO enrichment analysis

The biomaRt package (v. 2.40.5)^63^ was used to perform a GO term enrichment analysis. This package performs a statistical analysis to find out which BP or pathways are enriched with genes contained in the input clusters. Before analysis, all transcripts’ IDs were converted to the respective Entrez Gene identifiers and used as gene universe. The transcripts contained in the clusters used as input were also translated into genes. The database used as reference for gene annotation and mapping was the *Homo sapiens* genes (gGRCh38). In this context, the *p*-value refers to the probability of seeing a specific number of genes present in a concrete GO term, just by chance. The pathways recovered were the ones with a *p*-value equal or lower than 0.05, and with at least 3 genes from input present in the specific GO term. The process was repeated twice: once with the significantly up-regulated transcripts identified using the ANOVA-Dunnett’s tests and linear regression, and a second one with the significantly down-regulated genes, both after power filtering. Lastly, a False Discovery Rate (FDR) correction was calculated for the *p*-values of each cluster.

### PMI prediction modelling

A generalized linear model was implemented to predict PMI using Root Mean-Squared Error (RMSE) as the optimization parameter. The glmnet package (v. 3.0.1)^64^ was used to build a prediction model. Multiple values of alpha were tested after to generate the lowest RMSE. Cross-validation (CV) was applied as a validation technique and repetition was added to increase its robustness.

## Supplementary Information

Supplementary Information 1.

Supplementary Information 2.

Supplementary Information 3.

Supplementary Information 4.

Supplementary Information 5.

## Data Availability

The data discussed in this publication have been deposited in NCBI’s Gene Expression Omnibus (Edgar et al., 2002) and are accessible through GEO Series accession number GSE163207 (https://www.ncbi.nlm.nih.gov/geo/query/acc.cgi?acc=GSE163207).
