# Supplementary material for: Cell survival and DNA damage repair are promoted in the human blood thanatotranscriptome shortly after death
Source: Sci Rep. 2021 Aug 16;11:16585. doi: 10.1038/s41598-021-96095-z (PMC8368024; doi:10.1038/s41598-021-96095-z)
Supplement: Supplementary file 1 — Supplementary Information 1. [file 41598_2021_96095_MOESM1_ESM.pdf]

- **Spreadsheet 1**: contains all ANOVA-Dunnett’s test significant transcripts, after power filtering (335) ordered by increasing *p-value*. 227 were included in the up-regulated cluster (sheet 1). And 108 transcripts were included in the down-regulated cluster (sheet 2).
- **Spreadsheet 2**: contains all significant transcripts identified with linear regression, after power filtering (964) ordered by increasing *p-value.* The up-regulated cluster contains 499 transcripts (sheet 1). The down-regulated cluster contains 465 transcripts (sheet 2).
- **Spreadsheet 3**: contains the ANOVA-Dunnett’s test results between increasing bins (e.g.: 1 vs 2-6, 1-2 vs 3-6, 1-3 vs 4-6 , 1-4 vs 5-6 and 1-5 vs 6). The number of significant genes for each comparison were 1247, 1364, 1142, 2155 and 1646, respectively. Each comparison is saved in a different sheet and all transcripts are ordered by increasing *p-value.*
