# Supplementary material for: Cell survival and DNA damage repair are promoted in the human blood thanatotranscriptome shortly after death
Source: Sci Rep. 2021 Aug 16;11:16585. doi: 10.1038/s41598-021-96095-z (PMC8368024; doi:10.1038/s41598-021-96095-z)
Supplement: Supplementary file 5 — Supplementary Information 5. [file 41598_2021_96095_MOESM5_ESM.pdf]

Supplementary Material

Cell survival and DNA damage repair are promoted in the human blood thanatotranscriptome shortly after death

Laura G. Antiga[1,2,+], Lode Sibbens[1,+], Yasmina Abakkouy[1], Ronny Decorte[1,3], Wouter Van Den Bogaert[1,3], Wim Van de Voorde[1,3], and Bram Bekaert[1,3,*]

[1]KU Leuven, Forensic Biomedical Sciences, Department of Imaging & Pathology, Leuven, Belgium
[2]Universitat Pompeu Fabra (UPF), Departament de Ciències Experimentals i de la Salut (CEXS)
[3]UZ Leuven, Laboratory of Forensic Genetics and Molecular Archaeology, Leuven, Belgium
[*]Corresponding author: Bram Bekaert; KU Leuven, Forensic Biomedical Sciences, Herestraat 49 - box 7003 71, 3000 Leuven, Belgium; E-mail: bram.bekaert@kuleuven.be
[+]these authors contributed equally to this work

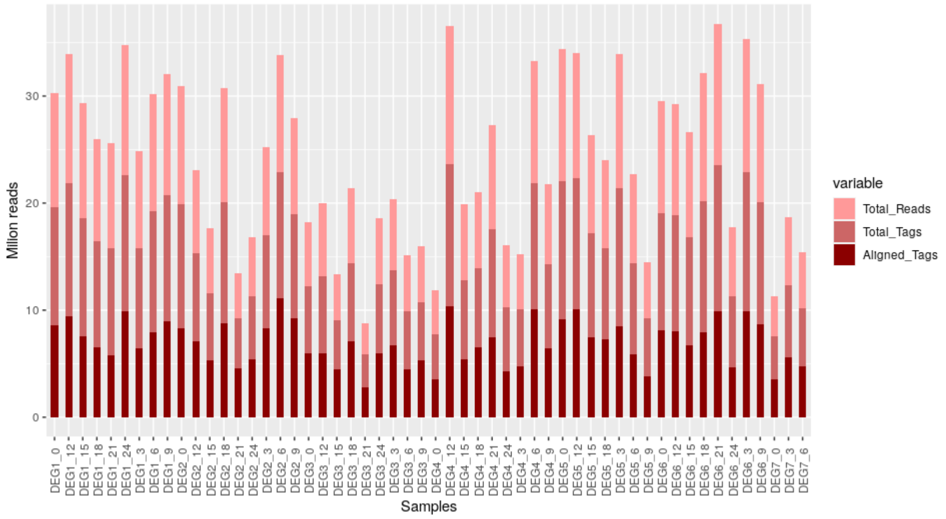

Figure S1. Sequencing depth in terms of million reads per sample. Figure shows the initial number of reads (Total_Reads), the relevant reads for the STAR algorithm (Total_Tags) and the amount of tags that were mapped to the reference genome (Aligned_Tags).

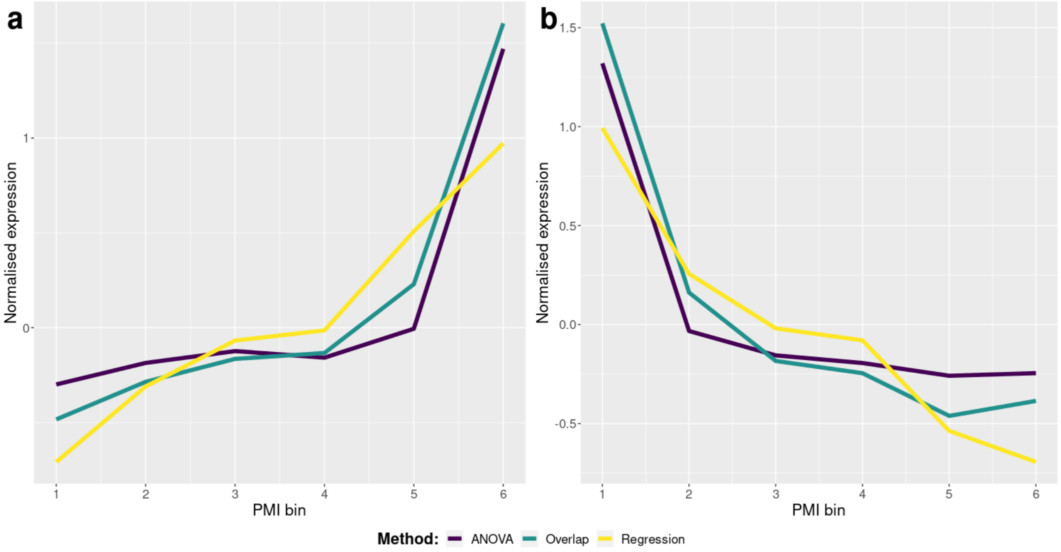

Figure S2. Mean expression of the significant transcripts found in the **a.** up-regulated cluster (n = 99) and **b.** down-regulated cluster (n = 89) both with ANOVA-Dunnett's test, regression analysis and the overlap between both methods.

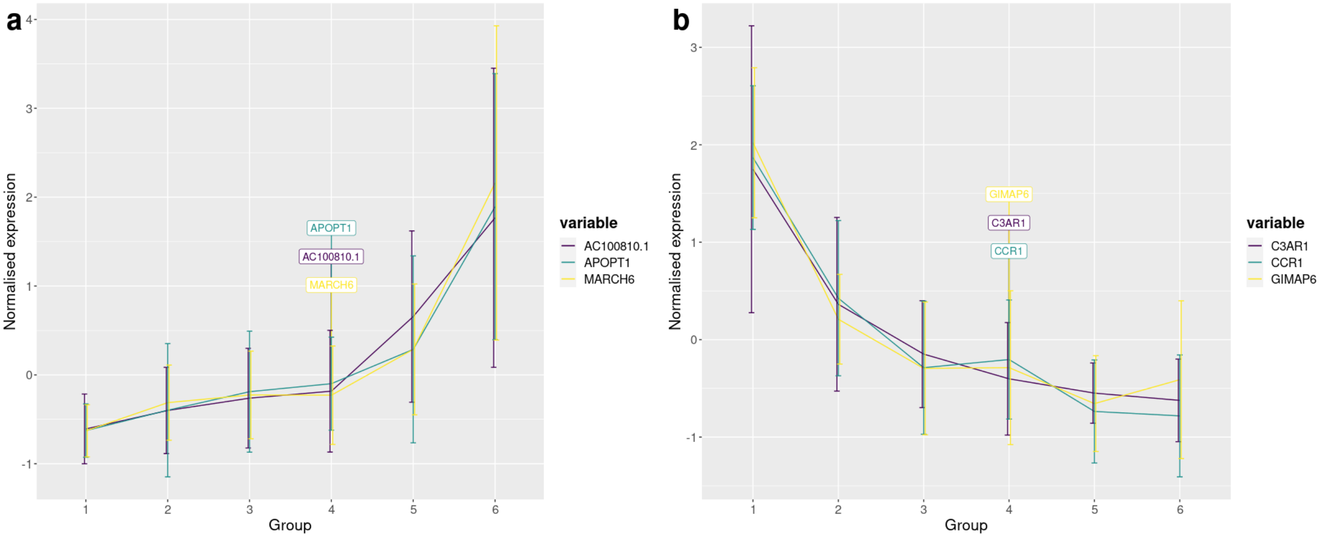

Figure S3. Mean expression, per bin, of the Top 3 significant genes found with ANOVA-Dunnett's test and regression analysis. **a.** in the up-regulated cluster and **b.** in the down-regulated cluster.

Table S1. Significant up-regulated genes using both ANOVA-Dunnett's test and linear regression analysis, after power filtering (n = 99)

Gene ID	Gene Name	p-value
ENSG00000253982	AC100810.1	1.3605×10^{-6}
ENSG00000145495	MARCH6	4.4447×10^{-6}
ENSG00000256053	APOPT1	1.0495×10^{-5}
ENSG00000145354	CISD2	1.8102×10^{-5}
ENSG00000133112	TPT1	2.1984×10^{-5}
ENSG00000144713	RPL32	2.8411×10^{-5}
ENSG00000155090	KLF10	3.7676×10^{-5}
ENSG00000197111	PCBP2	4.2062×10^{-5}
ENSG00000181704	YIPF6	5.8839×10^{-5}
ENSG00000186063	AIDA	5.9426×10^{-5}
ENSG00000176407	KCMF1	6.6883×10^{-5}
ENSG00000119782	FKBP1B	6.8476×10^{-5}
ENSG00000076043	REXO2	1.1697×10^{-4}
ENSG00000152484	USP12	1.3858×10^{-4}
ENSG00000198176	TFDP1	1.4082×10^{-4}
ENSG00000164237	CMBL	1.5854×10^{-4}
ENSG00000134779	TPGS2	1.6038×10^{-4}
ENSG00000180425	C11orf71	1.8767×10^{-4}
ENSG00000127540	UQCR11	1.9390×10^{-4}
ENSG00000114648	KLHL18	2.0615×10^{-4}
ENSG00000213639	PPP1CB	2.3226×10^{-4}
ENSG00000115317	HTRA2	2.4807×10^{-4}
ENSG00000114784	EIF1B	2.5743×10^{-4}
ENSG00000170881	RNF139	3.0057×10^{-4}
ENSG00000154114	TBCEL	3.6166×10^{-4}
ENSG00000205531	NAP1L4	3.6471×10^{-4}
ENSG00000119950	MXI1	4.1632×10^{-4}
ENSG00000154917	RAB6B	4.3940×10^{-4}
ENSG00000038210	PI4K2B	4.6901×10^{-4}
ENSG00000256338	RPL41P2	4.7881×10^{-4}
ENSG00000236810	ELOA-AS1	4.8158×10^{-4}
ENSG00000243317	STMP1	4.9509×10^{-4}
ENSG00000187479	C11orf96	5.4108×10^{-4}
ENSG00000122741	DCAF10	5.5230×10^{-4}
ENSG00000167705	RILP	5.7318×10^{-4}
ENSG00000115993	TRAK2	5.9417×10^{-4}
ENSG00000100387	RBX1	6.1646×10^{-4}
ENSG00000117614	SYF2	6.4612×10^{-4}
ENSG00000113328	CCNG1	6.9279×10^{-4}
ENSG00000138709	LARP1B	7.0320×10^{-4}
ENSG00000165113	GKAP1	7.1426×10^{-4}
ENSG00000177565	TBL1XR1	7.3754×10^{-4}
ENSG00000260592	AC130456.3	7.6070×10^{-4}
ENSG00000187097	ENTPD5	7.6298×10^{-4}
ENSG00000105926	MPP6	7.6312×10^{-4}
ENSG00000113312	TTC1	7.7271×10^{-4}
ENSG00000047597	XK	8.5294×10^{-4}
ENSG00000185630	PBX1	8.9937×10^{-4}
ENSG00000170315	UBB	9.1138×10^{-4}
ENSG00000182899	RPL35A	9.1569×10^{-4}
ENSG00000137601	NEK1	9.5494×10^{-4}
ENSG00000244313	AC024293.1	1.0742×10^{-3}
ENSG00000071967	CYBRD1	1.0979×10^{-3}
ENSG00000100490	CDKL1	1.2313×10^{-3}
ENSG00000110330	BIRC2	1.3344×10^{-3}
ENSG00000019995	ZRANB1	1.3649×10^{-3}
ENSG00000148341	SH3GLB2	1.4480×10^{-3}
ENSG00000118816	CCNI	1.4886×10^{-3}
ENSG00000126756	UXT	1.5081×10^{-3}
ENSG00000060237	WNK1	1.6762×10^{-3}
ENSG00000221983	UBA52	1.6930×10^{-3}
ENSG00000103502	CDIPT	1.6952×10^{-3}
ENSG00000137210	TMEM14B	1.7676×10^{-3}
ENSG00000198162	MAN1A2	1.7804×10^{-3}
ENSG00000100614	PPM1A	1.8123×10^{-3}
ENSG00000117394	SLC2A1	1.8417×10^{-3}
ENSG00000165669	FAM204A	1.9114×10^{-3}
ENSG00000200156	RNU5B-1	1.9268×10^{-3}
ENSG00000141179	PCTP	1.9279×10^{-3}
ENSG00000159346	ADIPOR1	1.9848×10^{-3}
ENSG00000125534	PPDPF	1.9884×10^{-3}
ENSG00000135045	C9orf40	2.1996×10^{-3}
ENSG00000198836	OPA1	2.2004×10^{-3}
ENSG00000110700	RPS13	2.2340×10^{-3}
ENSG00000164849	GPR146	2.4559×10^{-3}
ENSG00000234268	AP000936.3	2.5308×10^{-3}
ENSG00000189043	NDUFA4	2.5447×10^{-3}
ENSG00000099785	MARCH2	2.5484×10^{-3}
ENSG00000112146	FBXO9	2.6483×10^{-3}
ENSG00000180628	PCGF5	2.7785×10^{-3}
ENSG00000111666	CHPT1	2.7949×10^{-3}
ENSG00000119640	ACYP1	2.8058×10^{-3}
ENSG00000141030	COPS3	3.0503×10^{-3}
ENSG00000279088	AC022400.7	3.0835×10^{-3}
ENSG00000123106	CCDC91	3.1934×10^{-3}
ENSG00000115947	ORC4	3.2244×10^{-3}
ENSG00000163818	LZTFL1	3.2500×10^{-3}
ENSG00000044459	CNTLN	3.4109×10^{-3}
ENSG00000123933	MXD4	3.4712×10^{-3}
ENSG00000169018	FEM1B	3.5114×10^{-3}
ENSG00000203880	PCMTD2	3.5662×10^{-3}
ENSG00000156482	RPL30	3.6478×10^{-3}
ENSG00000115484	CCT4	3.6507×10^{-3}
ENSG00000237676	RPL30P4	3.7081×10^{-3}
ENSG00000112335	SNX3	3.7999×10^{-3}
ENSG00000143612	C1orf43	3.8352×10^{-3}
ENSG00000158578	ALAS2	3.9620×10^{-3}
ENSG00000013561	RNF14	3.9906×10^{-3}
ENSG00000197993	KEL	4.0990×10^{-3}

Table S2. Significant down-regulated genes using ANOVA-Dunnett's test and linear regression analysis, after power filtering (n= 89)

Gene ID	Gene Name	p-value	Gene ID	Gene Name	p-value
ENSG00000163823	CCR1	3.8423×10^{-9}	ENSG00000168282	MGAT2	3.3882×10^{-4}
ENSG00000133561	GIMAP6	1.5570×10^{-7}	ENSG00000146094	DOK3	3.7994×10^{-4}
ENSG00000171860	C3AR1	1.8524×10^{-7}	ENSG00000128604	IRF5	3.9179×10^{-4}
ENSG00000133313	CNDP2	2.2824×10^{-7}	ENSG00000100368	CSF2RB	4.1987×10^{-4}
ENSG00000095370	SH2D3C	4.8757×10^{-7}	ENSG00000169032	MAP2K1	4.4437×10^{-4}
ENSG00000101916	TLR8	5.7184×10^{-7}	ENSG00000163694	RBM47	4.4994×10^{-4}
ENSG00000172936	MYD88	1.5361×10^{-6}	ENSG00000107862	GBF1	4.6726×10^{-4}
ENSG00000166927	MS4A7	3.5344×10^{-6}	ENSG00000179051	RCC2	4.8665×10^{-4}
ENSG00000165025	SYK	3.6393×10^{-6}	ENSG00000131043	AAR2	5.1639×10^{-4}
ENSG00000168329	CX3CR1	4.4720×10^{-6}	ENSG00000120949	TNFRSF8	6.5179×10^{-4}
ENSG00000185009	AP3M1	4.7967×10^{-6}	ENSG00000185215	TNFAIP2	6.5906×10^{-4}
ENSG00000145416	MARCH1	4.9663×10^{-6}	ENSG00000140598	EFL1	6.7003×10^{-4}
ENSG00000162739	SLAMF6	6.1630×10^{-6}	ENSG00000143337	TOR1AIP1	6.7653×10^{-4}
ENSG00000119669	IRF2BPL	8.5538×10^{-6}	ENSG00000165879	FRAT1	7.9735×10^{-4}
ENSG00000121297	TSHZ3	9.0497×10^{-6}	ENSG00000136536	MARCH7	8.1177×10^{-4}
ENSG00000118217	ATF6	1.0178×10^{-5}	ENSG00000088298	EDEM2	8.1229×10^{-4}
ENSG00000206337	HCP5	1.1308×10^{-5}	ENSG00000205423	CNEP1R1	8.7217×10^{-4}
ENSG00000168040	FADD	1.4194×10^{-5}	ENSG00000135090	TAOK3	9.6578×10^{-4}
ENSG00000064012	CASP8	1.7139×10^{-5}	ENSG00000129450	SIGLEC9	9.7356×10^{-4}
ENSG00000111817	DSE	1.8724×10^{-5}	ENSG00000163848	ZNF148	1.0284×10^{-3}
ENSG00000272941	AC083862.2	2.0403×10^{-5}	ENSG00000167528	ZNF641	1.1161×10^{-3}
ENSG00000188305	PEAK3	2.9354×10^{-5}	ENSG00000100403	ZC3H7B	1.1522×10^{-3}
ENSG00000262758	CTD-3195I5.	2.9672×10^{-5}	ENSG00000131759	RARA	1.2196×10^{-3}
ENSG00000170837	GPR27	3.0278×10^{-5}	ENSG00000169105	CHST14	1.3156×10^{-3}
ENSG00000110777	POU2AF1	3.4312×10^{-5}	ENSG00000163513	TGFBR2	1.3604×10^{-3}
ENSG00000169508	GPR183	4.2424×10^{-5}	ENSG00000145868	FBXO38	1.5187×10^{-3}
ENSG00000198130	HIBCH	4.3453×10^{-5}	ENSG00000136867	SLC31A2	1.6028×10^{-3}
ENSG00000146592	CREB5	4.3872×10^{-5}	ENSG00000224870	AL391244.1	1.6914×10^{-3}
ENSG00000181631	P2RY13	4.4689×10^{-5}	ENSG00000164086	DUSP7	1.7338×10^{-3}
ENSG00000109320	NFKB1	4.9293×10^{-5}	ENSG00000189337	KAZN	1.8819×10^{-3}
ENSG00000196329	GIMAP5	5.2487×10^{-5}	ENSG00000127954	STEAP4	1.9197×10^{-3}
ENSG00000171049	FPR2	6.1326×10^{-5}	ENSG00000137275	RIPK1	2.0328×10^{-3}
ENSG00000104228	TRIM35	7.0599×10^{-5}	ENSG00000172716	SLFN11	2.4438×10^{-3}
ENSG00000138172	CALHM2	7.6383×10^{-5}	ENSG00000185033	SEMA4B	2.4646×10^{-3}
ENSG00000221869	CEBPD	8.5732×10^{-5}	ENSG00000171700	RGS19	2.5159×10^{-3}
ENSG00000101347	SAMHD1	1.2410×10^{-4}	ENSG00000132718	SYT11	2.7065×10^{-3}
ENSG00000174718	KIAA1551	2.0112×10^{-4}	ENSG00000174125	TLR1	2.8260×10^{-3}
ENSG00000235750	KIAA0040	2.0317×10^{-4}	ENSG00000139318	DUSP6	2.9468×10^{-3}
ENSG00000160791	CCR5	2.0518×10^{-4}	ENSG00000166289	PLEKHF1	3.0050×10^{-3}
ENSG00000241878	PISD	2.1815×10^{-4}	ENSG00000157593	SLC35B2	3.0058×10^{-3}
ENSG00000106066	CPVL	2.4019×10^{-4}	ENSG00000142867	BCL10	3.0076×10^{-3}
ENSG00000112796	ENPP5	2.6451×10^{-4}	ENSG00000064313	TAF2	3.6151×10^{-3}
ENSG00000115207	GTF3C2	2.8620×10^{-4}	ENSG00000165609	NUDT5	3.8118×10^{-3}
ENSG00000166189	HPS6	3.1078×10^{-4}	ENSG00000136826	KLF4	4.0492×10^{-3}
ENSG00000106780	MEGF9	3.3837×10^{-4}			
